# Supplementary material for: Interictal magnetoencephalography abnormalities to guide intracranial electrode implantation and predict surgical outcome
Source: Brain Commun. 2023 Oct 25;5(6):fcad292. doi: 10.1093/braincomms/fcad292 (PMC10636564; doi:10.1093/braincomms/fcad292)
Supplement: fcad292_Supplementary_Data [file fcad292_supplementary_data.docx]

# Supplementary

# Surgical outcome separability of the $\boldsymbol{D}_{\boldsymbol{RS}}$

The $D_{RS}$ is a measure that was recently shown to relate to post-operative seizure freedom in cohorts of individuals with refractory epilepsy. We compute the MEG and iEEG $D_{RS}$ values for the cohort of 32 individuals with refractory neocortical epilepsy using only tissue that has coverage in both modalities. The results across the cohort, Supplementary Figure [1](#fig:DRS_results) demonstrate the separability of surgical outcome groups based on the derived $D_{RS}$ scores. The $D_{RS}$ for both modalities performs well and in the hypothesised direction, separating surgical outcome groups with an AUC$>0.7$.


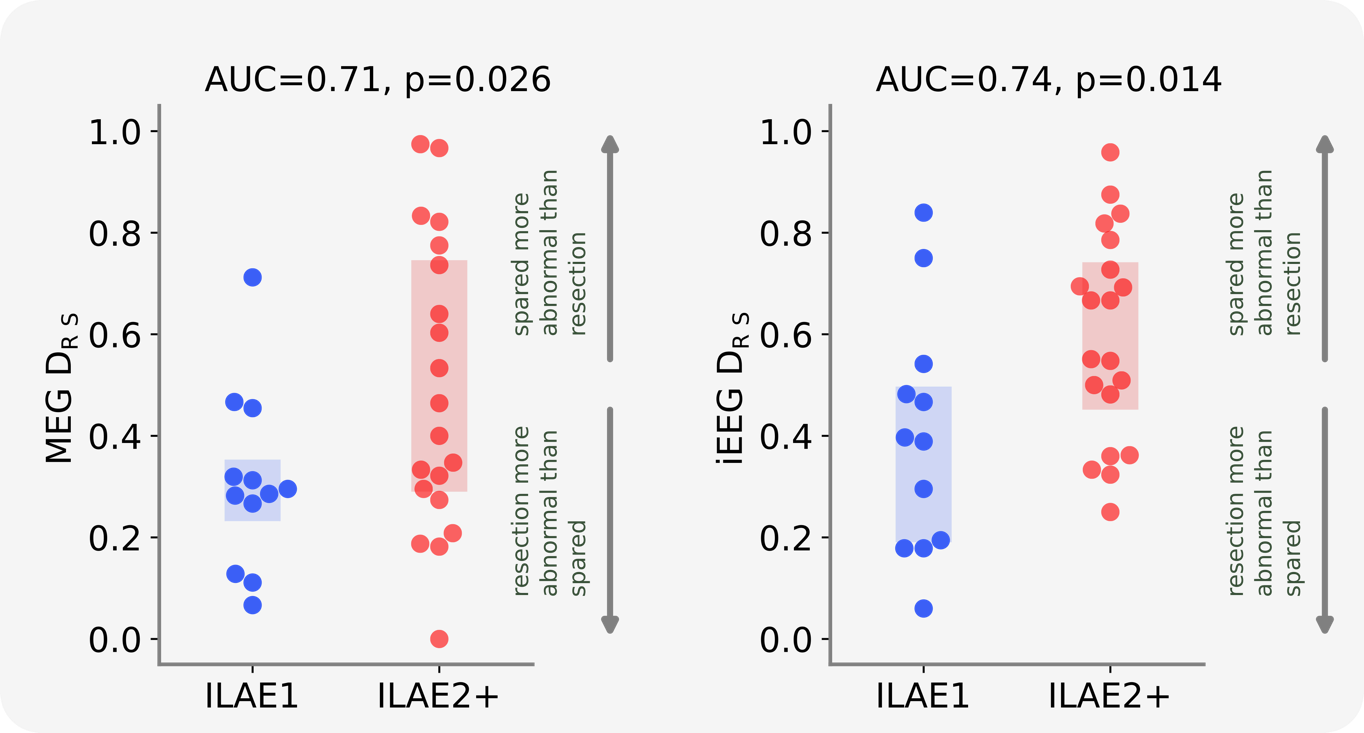


**Supplementary Figure 1: Surgical outcome separability of the MEG and iEEG** $D_{RS}$ **measures**. Boxplots illustrated the differences between surgical outcome groups based on the corresponding $D_{RS}$ scores. Each point corresponds to an individual, blue points correspond to seizure-free individuals, and red points to non-seizure-free individuals. Both measures of $D_{RS}$ separate surgical outcome groups well and in the hypothesised direction. That is, with smaller $D_{RS}$ values attributed to seizure-free patients as the most abnormal tissue has been resected. Statistical significance of the AUC was calculated using a one-tailed Mann-Whitney U test.

**Scanning the threshold of resection**

We replicated our analysis but varied the threshold of pre- and post-operative volume change required for a region to be defined as resected. In this subsequent analysis the definition of resection and threshold was consistent across modalities. Supplementary Figure 2 illustrates the results of our analysis and demonstrates that regardless of threshold the performance of our measures and model are good, with the best performing model always pertaining the to the nomogram which combines all three measures together.


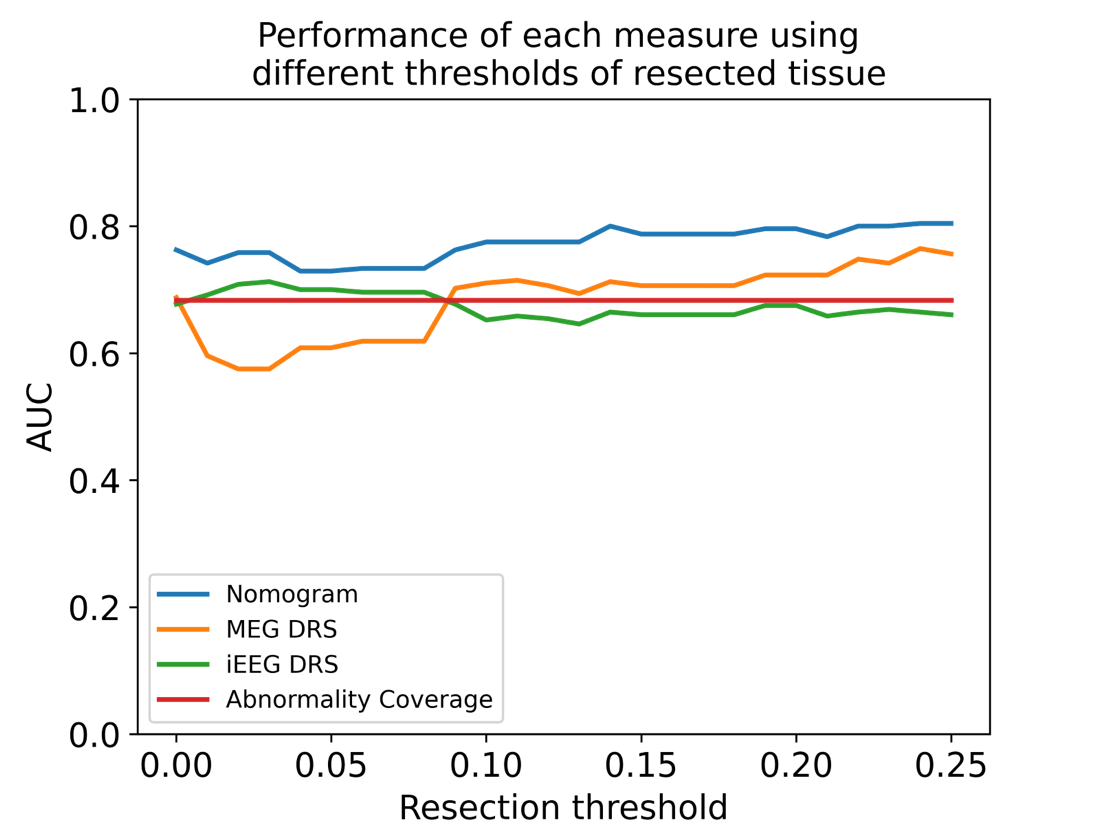


***Supplementary Figure 2: Analysis using different thresholds to define resected tissue.*** *In this analysis the definition of resected and spared is consistent across modalities. The three individual markers, abnormality coverage (red), iEEG DRS (green) and MEG DRS (orange) are shown for thresholds ranging between 0 and 0.25. Moreover, the results of the combined model, the nomogram (blue) are also visualised.*

# Table of patient data

Patient information can be found on Supplementary Table 1.

Supplementary table 1: Abbreviations: FTBTC: Focal to bilateral tonic clonic seizures. NA: Not Available. DNT: dysembryoplastic neuroepithelial tumor. G/GL: Glioma. FCD: Focal cortical dysplasia. TSL: Tuberous sclerosis.


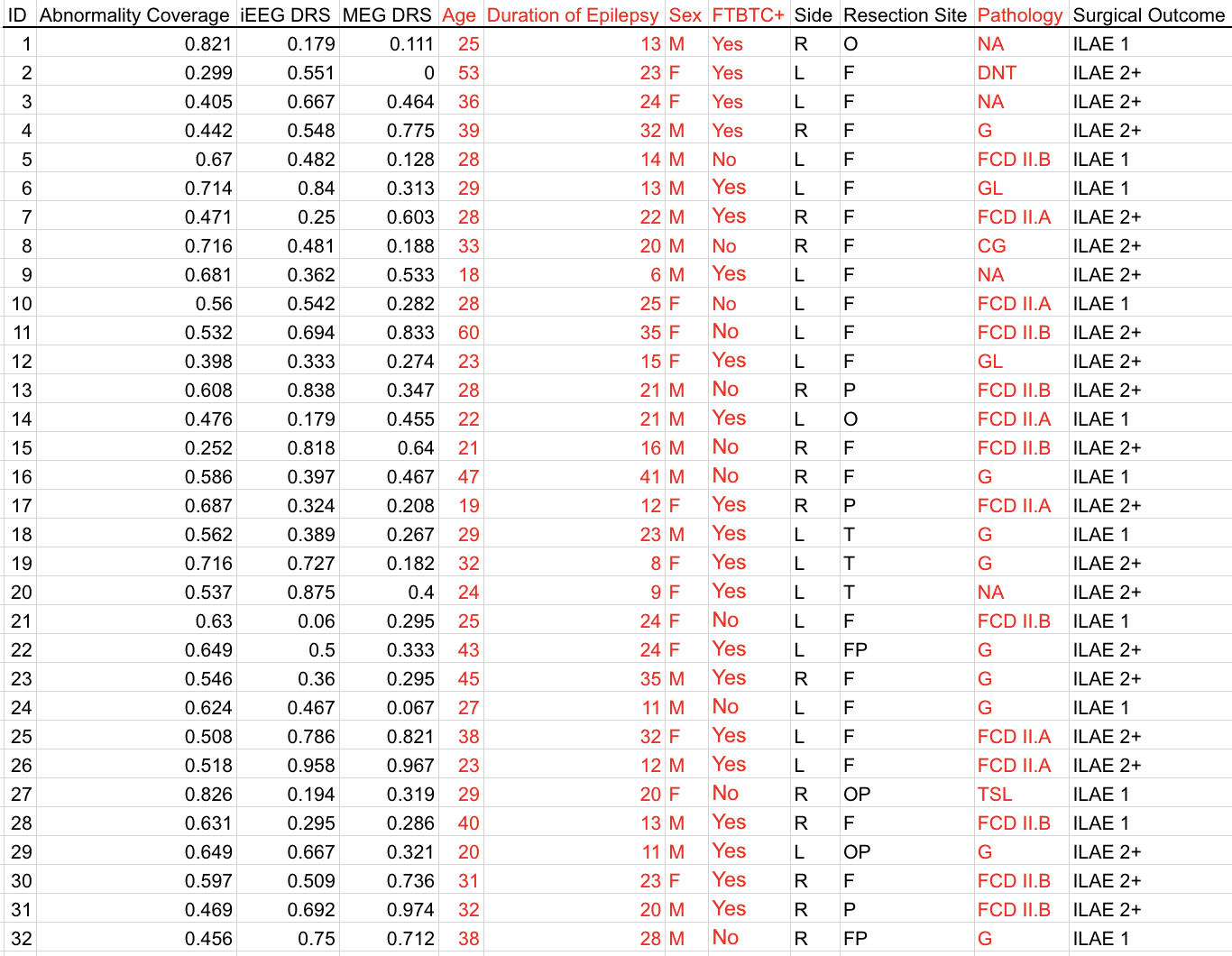


# Table of results using each individual frequency band in isolation

|  | Abnormality Coverage | MEG DRS | EEG DRS | Nomogram |
| --- | --- | --- | --- | --- |
| Delta | 0.63 | 0.72 | 0.75 | 0.79 |
| Theta | 0.46 | 0.37 | 0.59 | 0.68 |
| Alpha | 0.69 | 0.62 | 0.55 | 0.70 |
| Beta | 0.59 | 0.56 | 0.73 | 0.75 |
| Gamma | 0.56 | 0.44 | 0.71 | 0.71 |
| Maximum | 0.68 | 0.71 | 0.74 | 0.80 |
